# Hydrodynamic drag force on a sphere approaching a liquid-liquid interface

**DOI:** 10.1016/j.heliyon.2020.e04089

**Published:** 2020-05-31

**Authors:** K.C. Nduaguba, J.L. Chukwuneke, S.N. Omenyi

**Affiliations:** Mechanical Engineering Department, Nnamdi Azikiwe University, Awka, Nigeria

**Keywords:** Chemical engineering, Mechanical engineering, Materials science, Materials application, Materials mechanics, Hydrodynamic force, Drag force, Rigid sphere, External-internal diameter, Liquid-liquid interface

## Abstract

The difficulties involved in trying to model the motion of a solid particle through surfaces, particularly at the liquid-liquid interface, are mainly due to the continuous deformation of the surface, not only as the particle progresses through the surface, but also before its penetration into the lower liquid. This study investigated experimentally and theoretically, the hydrodynamic drag force on a sphere approaching a liquid-liquid interface. The experiment ball material of steel of different ball diameters ranging from 1.5E-3 to 8.69E-3m in four immiscible liquids of distilled water, kerosene, glycerol and engine oil of densities; 1000 kg/m^3^, 820 kg/m^3^, 1260 kg/m^3^ and 848.3 kg/m^3^ respectively, were considered. The drop either penetrated the interface without opposition, or spent some time at the interface before penetrating, or it remained at interface maintain a certain interface curvature. The mathematical model of the resulting velocities as a function of the size ratio R/R∗ was obtained. The Stinson and Jeffry technique was modified in the theoretical analysis (one ball internal to the other - the larger ball providing curved surface at contact) and using MATLAB algorithm obtained the correction factor to the velocity and hence the hydrodynamic drag force was obtained. The model mathematical equation for the velocity was found comparable to those obtained experimentally. The hydrodynamic drag forces calculated theoretically and experimentally were further analyzed using ANOVA for same size ratio R/R∗ of 0.83. It was found that for steel balls, the experimental and theoretical results are significantly the same confirming the validity of the mathematical model and this work. This kind of study is valuable in biomechanics in the area of blood flow in arteries and capillaries. It is also important in determining the motion of small particles or macromolecules near permeable surfaces, and determining particle deposits on reverse osmosis, mineral filtration, and dialysis or drip irrigation surfaces.

## 1 Introduction

A number of manufacturing processes, such as sediment transport and deposition in pipelines, alluvial channels, chemical engineering and powder processing, provide a description of the motions of the immersed bodies in fluids. A particle that falls or rolls a plane in a fluid under the influence of gravity will accelerate until the resistance forces, including buoyancy and drag, balance the gravitational force (Datta and Srivastava, 2000; Datta and Pandya, 2001; Sauvagya, 2013). The constant velocity attained is called terminal/settling velocity at that stage. In many industrial applications, knowledge of the terminal velocity of liquid solids is required: mineral processing, hydraulic transport of coal and ore slurry systems, solid-liquid mixing, fluidizing equipment, thickeners, oil and gas drilling, and even geothermal drilling (Andrew et al., 2007; Sauvagya, 2013; Loudet et al., 2020). When the fluid is forced through the tube, the particles that make up the fluid generally move faster near the axis of the tube and more slowly near its walls; therefore, some stress (pressure difference between the two ends of the tube) is needed to overcome the friction between the layers of the particles to keep the fluid moving (Ryu and Owen, 2005; Ashmawy, 2011; Dani et al., 2015; Xingxun, 2015).

The considerable physical interest in interface science is the understanding of the mechanism by which a solid particle can penetrate a free surface or an interface between immiscible fluids (Arbaret et al., 2011; Mousazadeh et al., 2018). The difficulties experienced in trying to model the motion of a particle through such surfaces are many and mainly attributable to the continuous deformation of the surface, not only as the particle progresses through the surface, but also prior to its penetration (Elio, 2017; Dietrich et al., 2011). This means that an accurate theoretical description of the mechanism would have to take into account the background of the motion of the particles as they approach the surface but such a complex theoretical solution would have to be determined numerically with the continuously deforming surface forming an unknown boundary problem (Mortazavi and Tryggvason, 2000; Marcello, 2008; Jenny and Dusek, 2009; Zhu, 2018). Elio (2017) stated that when a sphere is placed in an infinite, incompressible Newtonian fluid, such as oil and water (Dani et al., 2015), it initially accelerates due to gravity and, after a short transient time, the sphere reaches a steady flow rate (constant terminal velocity) and there will be no change in linear momentum for the velocity to be stable. This problem will need to be addressed both theoretically and experimentally.

According to Jalaal and Ganji (2010) the resistance drag force depends on the drag coefficient and the terminal velocity of the particles. Several attempts have been made to relate the drag coefficient to the Reynolds number (Andrew et al., 2007; Modo et al., 2017; Zhang, 2018) and most of these applications involve a description of the position, velocity and acceleration of the particles over time, and where it is often necessary to determine the trajectories of the acceleration of the particles in the fluid for design or improved operation (Jalaal and Ganji, 2010; Elio, 2017). To create a model for a sphere falling through one or two fluids in different containers, the relevant forces must be summed up and the resulting equations modified to make accurate predictions of the physical situation (Mendez, 2011; Srivastava, 2013). As the sphere passes through the fluid, several forces are acting on it. There is the obvious force of gravity (F_G_) that forces the sphere down through the fluid, there is also the force of drag (F_D_) that prevents the fall of the sphere and the force of buoyancy (F_B_) (Tropea et al., 2007). Sauvagya (2013) stated that of the several forces which affect the hydrodynamics of the Newtonian fluid between them, drag force and wall effect is prominent and that whenever there is a difference in velocity between the particle and its surrounding fluid, the fluid will exert a resistive force on the particle, either the fluid may be at rest and the particle may move through it or the particle may be a particle. Jenny et al. (2004) investigated the effects of a sphere falling/rising under the gravitational force of Newtonian fluids.

Much work has been done, as reported in the available literature, for spheres approaching plane surfaces as well as deformable surfaces. Exact solutions of terminal velocity of spheres falling through the deformable interface have been registered, and some of these forces have been properly provided with the correcting factor. It is against this backdrop that this study seeks to consider the same problem with a different approach to the perspective of considering two spheres moving in a viscous fluid, one internal to the other, with the idea of having an external sphere when it is large as providing a curved interface.

## 2 Mathematical model

### 2.1 Drag correction factor model

To model the motion of a solid sphere at a deformable interface, the method of Stimson and Jeffrey (1926) is used with some modification of the boundary conditions. These authors considered two spheres external to each other and in motion, but in this approach, the two spheres would be seen from one internal to the other. The mathematical model was developed using part of the Brenner (1961) method, which examined the slow motion of a sphere through a viscous fluid towards a plane surface using the results of Stimson and Jeffrey (1926). They ignored the deformation of the surface and assumed a constant clearance between the sphere and interface at the same velocity. Thus, the force is given by

(1) $$F_{z}=\pi \mu \int \rho^{3}\frac{\partial }{\partial n}\left(\frac{E^{2}\left(\varphi \right)}{\rho^{2}}\right)ds$$

The integral taken around the meridian section of the solid in a direction making a positive right angle with direction n, n = outward normal from solid, ρ is the distance from axis is given by

(2) $$E^{2}=\frac{\partial^{2}}{\partial r^{2}}+\frac{sin\theta }{r^{2}}.\frac{\partial }{\partial \theta }\left(\frac{1}{sin\theta }.\frac{\partial }{\partial \theta }\right)$$

The trial solution to Eq. (2) is

(3) $$\varphi =sin^{2}\theta F\left(r\right)$$

With this formulation, the force on the sphere can be calculated for given boundary conditions.

Take ζ and η as curvilinear coordinates in a meridian plane defined by the conformal transformation

(4) $$Z\pm i\rho =iacot\frac{1}{2}\left(\text{η }+\text{iζ}\right)$$

Equivalently, by Stimson and Jeffrey (1926):

(5) $$\text{ζ}+i\text{η}=\ln\frac{\rho +i\left(Z+a\right)}{\rho +i\left(Z-a\right)}$$

So,

(6) $$\rho =\text{a}\frac{\text{sinη}}{\text{coshζ}-\text{cosη}};z=\text{a}\frac{\text{ sinhζ}}{\text{coshζ}-\text{cosη}}$$

Rotating the curves ζ = constant about z-axis, one gets a family of spheres having z = 0 (ζ = 0) for a common radical plane (since ζ = 0, is a sphere of infinite radius which is equivalent to the entire plane z = 0). In the case of two spheres, one internal to the other will be defined by

(7) ζ=αandζ=β

(α>0, β>0 but α≫β where ζ=α is the smaller sphere) whileα, β and the constant c may be chosen so that these spheres have any radii and any centre distance given by the difference between their radii.

**Figure 1:**
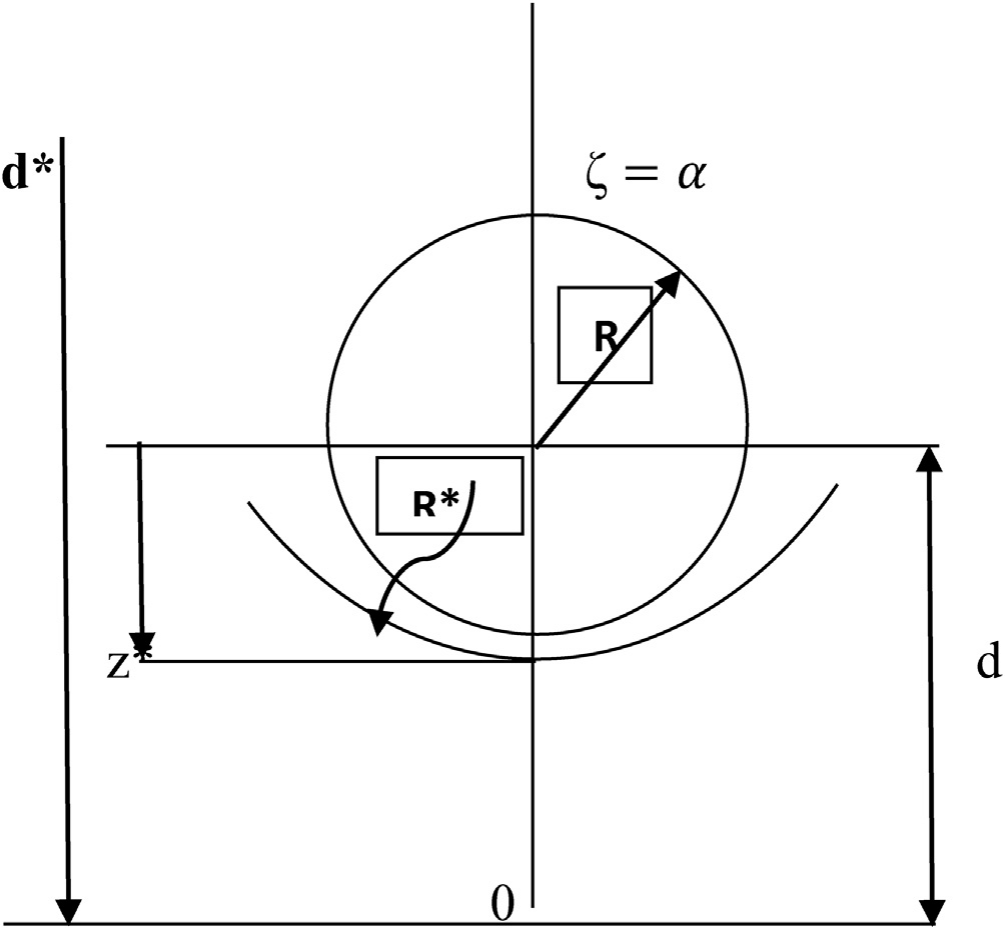
Sketch shows two spheres of radii R and R∗ one internal to the other (the bipolar coordinates ζ=α and ζ=β defines the two spheres).

The case where a sphere approaches a deformable interface can be visualized as the notion of a small sphere within a large sphere where the small sphere rests close to the inside of the lower part of a large sphere as shown in Figure (1). Let the radius of the small sphere be R and the radius of the large sphere which provides a large curvature on the interface be R∗. Modifying the signs of equality and inequality of the bipolar coordinate,

(8) ζ=α,ζ=β,(α>0,  β>0withα≫β )

Where the sphere ζ=α is internal to the sphere, ζ=β. The radii of the spheres and distances of their centres to the origin are given by (see Figure 1). If the spheres are of radii,Rand R∗ have their centres at distances d and d∗ from the same side of the origin, then

(9) $$\begin{array}{ll} R=a\;\cos\;echa, & R*=a\;\cos\;ech\beta \\ d=a\;\coth\;a, & d*=a\;\coth\;\beta \end{array}\}$$

Realize that for ζ = β = 0, a second sphere is a plane. The distance between the centre of the small sphere and the lower part of the larger sphere is;

(10) $$z^{*}=R^{*}+d-{\text{d}}^{\text{∗}}$$

Using Eq. (9) in Eq. (10);

(11) $$\frac{1}{\text{sinhα}}\left(\frac{z^{*}}{\text{R}}-\text{coshα}\right)=\frac{1}{\text{sinhβ}}\left(1-\text{coshβ}\right)$$

And

(12) $$\frac{R^{*}}{R}=\frac{sinh\alpha }{sinh\beta }$$

When α(α>0) is chosen arbitrarilyβ(β>0), will be determined so that β≪α, and $\frac{R^{*}}{R}\gg 1$, then $\frac{Z^{*}}{R}$ can be determined from Eq. (11).

The same solution as that obtained by Stimson and Jeffrey (1926) was applied. When R ∗ is very large and R small, the image would be very similar to that of a sphere placed on a deformed interface. Thus, the viscous drag force will be given by

(13) $$F_{D}=6\pi \mu RV\zeta$$

Where

(14) $$\zeta =1+\zeta^{*}$$

(15) $$\zeta^{*}=\frac{\sqrt{2sinh\alpha }}{3a^{2}V}\sum_{n=1}^{\infty }\left(2n+1\right)\left({\text{A}}_{\text{n}}-{\text{B}}_{\text{n}}+{\text{C}}_{\text{n}}-{\text{D}}_{\text{n}}\right)$$

The values of the constants A_n_, B_n_, C_n_ and D_n_ represents the boundary conditions on the spheres obtained by Stimson and Jeffrey (1926), k = viscosity. When these values are obtained in Eq. (15) and Eq. (14), Eq. (13) will be completely defined.

### 2.2 Method of solution

#### 2.2.1 Curve-fitting

The determination of zeta ζ involves the use of a high-speed computer program (MATLAB). The values of α were assumed between 0.05 and 1.0; then for a series of $\frac{\zeta }{\text{R}}$ values, Eq. (12) was solved for β and the values of $\frac{\zeta }{\text{R}}$ for which β < α, and β > 0, were recorded. Then $\frac{R^{*}}{R}$ was calculated for each pair of α and β from Eq. (12) with these pairs of values,ζ was calculated from Eq. (15). The terminating point used in the program was 3ζ=+or−0.001 and n in Eq. (15) was made to vary from 70 to 150 depending on relative values of α and β. From these results an explicit form Eq. (16) was chosen to ensure ζ does not become infinite, zero or negative as size ratios change.

(16) $$\zeta =\frac{A}{{\left(1-\frac{R}{z^{*}}\right)}^{\in }{\left(1-\frac{R}{R^{*}}\right)}^{∋}}$$

Where A,∈and∋ are constants.

## 3 Materials and methods

### 3.1 Materials and equipment

Suspending liquids for this experiment include; distilled water, automobile engine oil (Mobile Engine Oil, SAE 5W 30), and glycerol while the solid sphere is made of steel balls of five (5) different ball sample sizes. The Steel balls were made by crushing the car bearings in which the balls were mounted. The need for accurate measurement of the experimental samples led to a careful selection of the right equipment such as a digital Vernier calliper, digital scale, high-speed digital cameras, two calibrated 100ml cylindrical glass tubes, a pair of forceps and retort stand.

### 3.2 Methods

The experimental method was followed by the technique used by Abaid and Adalsteinsson (2004) who carried out an experiment involving the use of a glass tank of a given size, spread with salt in different density profiles. An array of spherical glass beads were released from the top of the tank and the motion was recorded. The camera was set at a distance from the tank and the height from the ground to the middle of the lens. The above method was used for this experiment, except that a calibrated cylindrical glass tube would replace a glass tank, while a different ball material would be used instead of a glass bead. During the experiment, two high-speed cameras were used.

**Figure 2:**
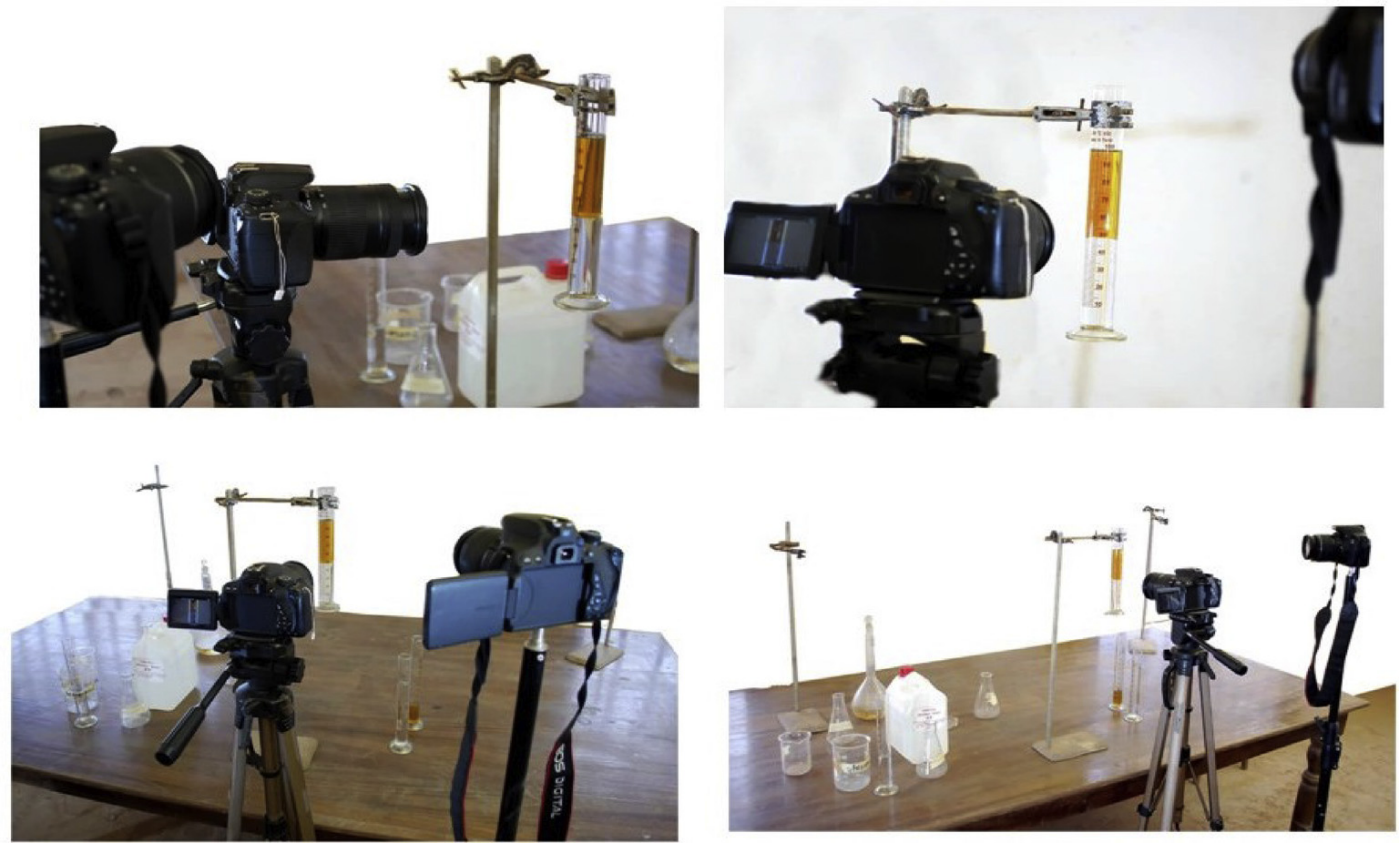
Shows the experimental setup in the laboratory.

The two-liquid experiment was carried out as follows; a two-100-ml cylindrical glass tube was filled with fluids for the test in combination with automobile engine oil and water; automobile engine oil and glycerol. The two liquids in each of the tubes had a 1:1 ratio of 50 ml–50 ml (see Figure 2). First, the denser fluid was poured into the glass tubes and then the glass tube was fixed to the retort stand. Liquids were allowed to settle for at least 15 min before each experiment. The two cameras used were positioned in such a way that one could capture the ball at the interface as it struggles with the hydrodynamic force that resists its passage into the second liquid and the other camera to capture the video of the entire operation. Canon cameras were used to capture the entire process and the actions of the ball at the liquid-liquid interface.

**Figure 3:**
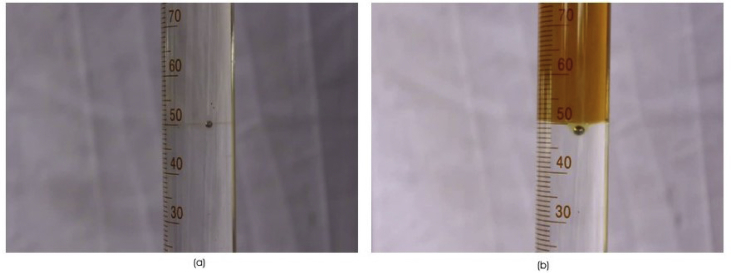
Shows liquid-liquid Steel Ball Interfacial Interaction (a) Kerosene-Glycerol-Steel Ball (b) Engine Oil-Water-Steel Ball.

The white background was used for a better view and the laboratory was protected from interruptions during the experiment. The diameters of the balls and glass tubes used in the experiment were measured using a digital Vernier calliper; the mass was measured using a digital weighing scale and the length of the tube was measured using a meter law. The balls were slowly placed on the surface of the first liquid, making slight surface contact with the first liquid before the ball was released. The whole process was then recorded using the two cameras provided. The use of balls that were much smaller than the container helped to neglect the wall effects of the sides of the cylindrical tube on the motion of the balls during the experiment (see Figure 3). For the two-fluid experiments, every movement of the ball was captured starting from the first liquid to the interface and finally to the bottom of the tube. Each image was properly analyzed to obtain the required accurate velocity of the fall of the sphere by measuring the distance dropped with the time taken for the fall. The same method was used with all ball sizes and with different fluids to complete the experiment.

## 4 Results and discussion

### 4.1 Experimental results

**Figure 4:**
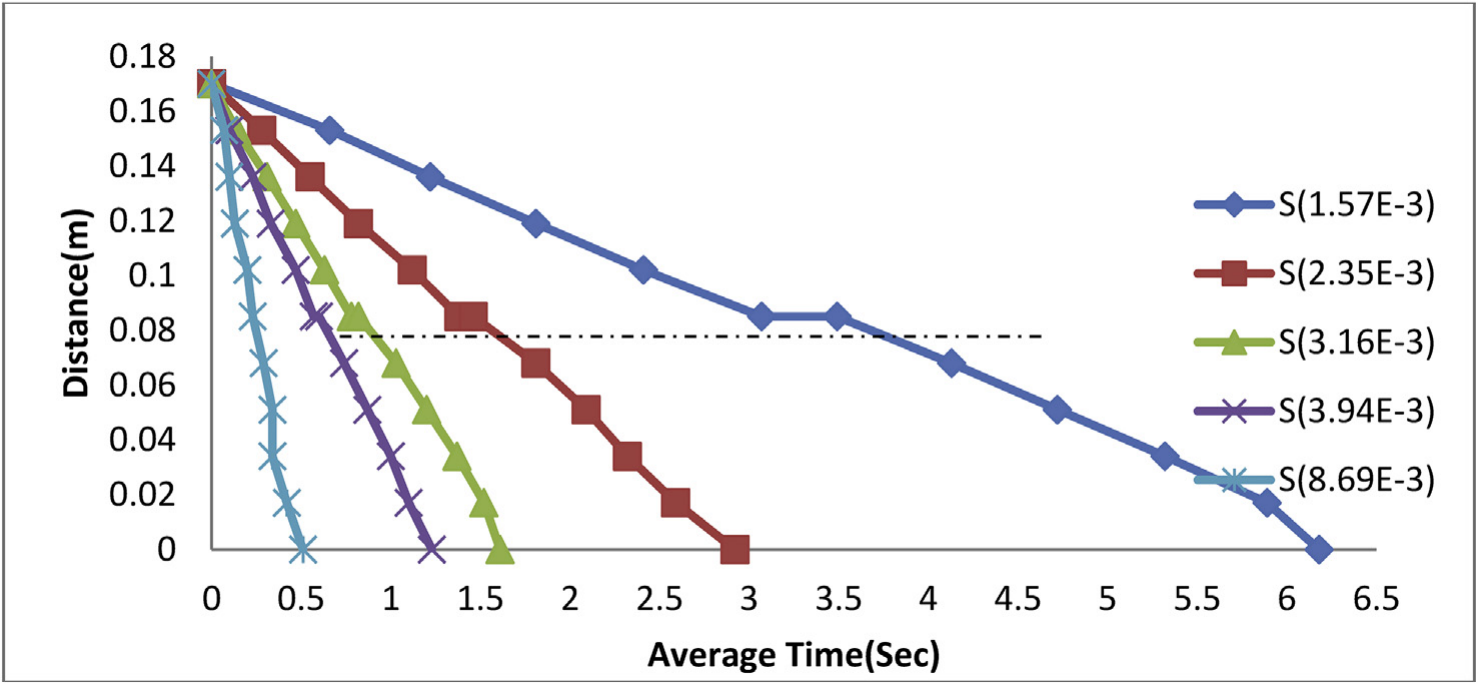
Plot of Distance against Average Time of steel balls in Engine Oil-Glycerol Medium.

**Figure 5:**
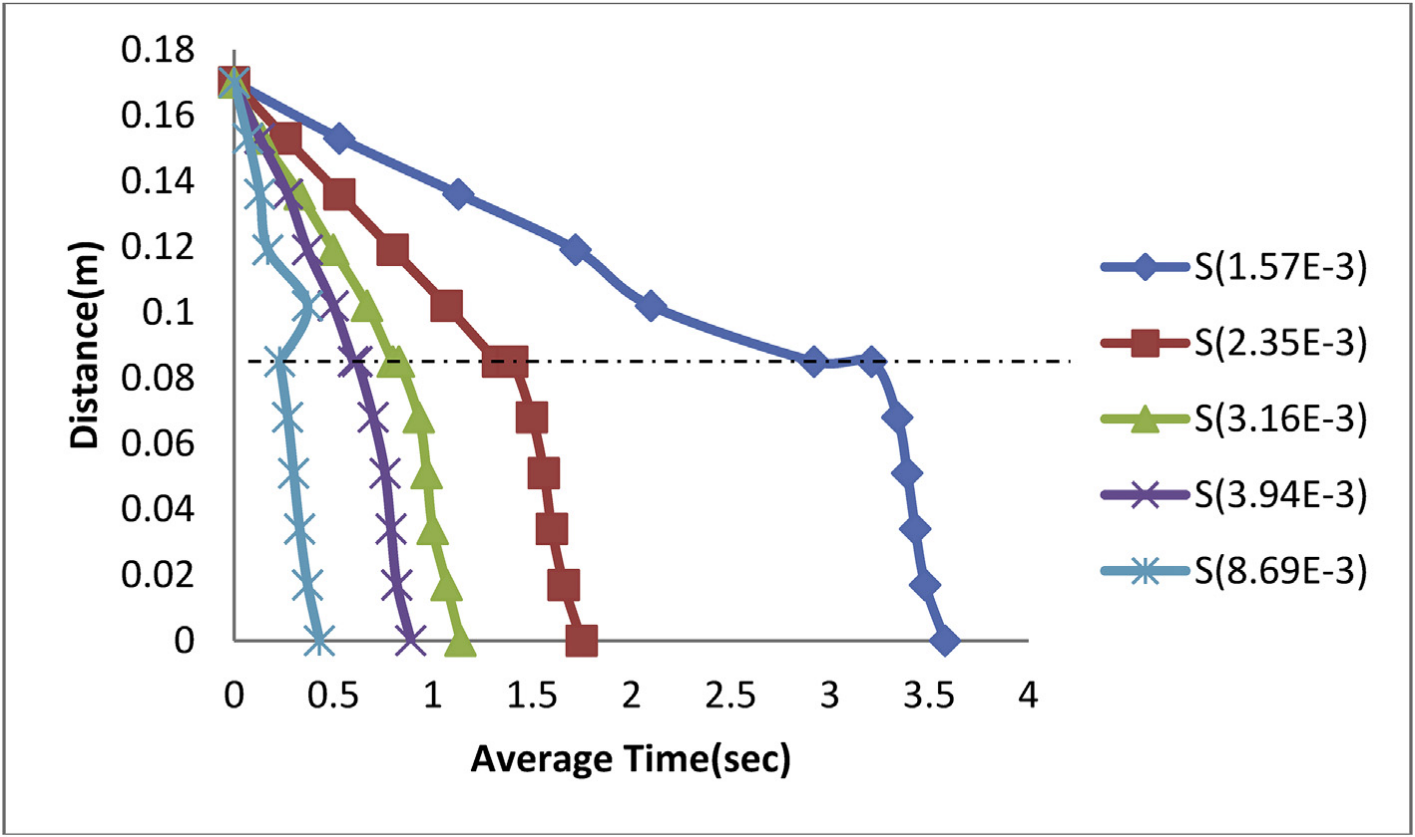
Plot of Distance against Average Time of steel balls in Engine Oil-water Medium.

**Figure 6:**
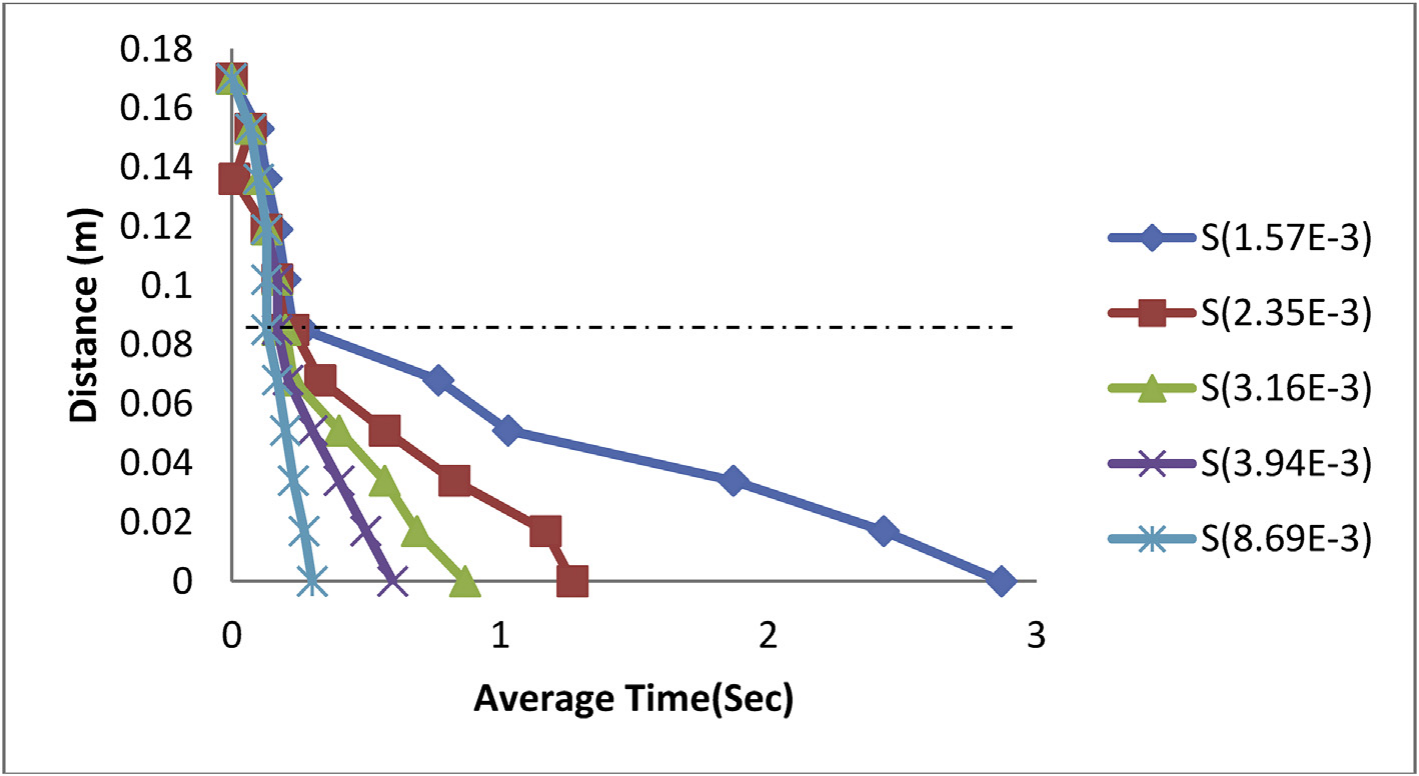
Plot of Distance against Average Time of steel balls in Kerosene-Glycerol Medium.

**Figure 7:**
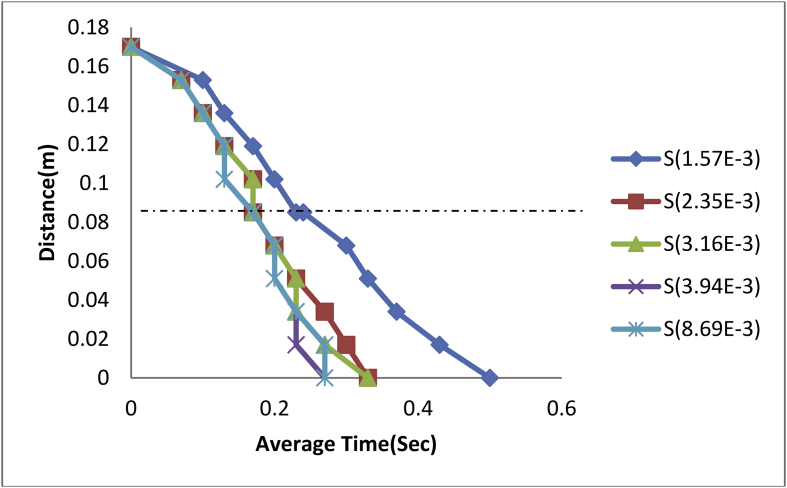
Plot of Distance against Average Time of steel balls in Kerosene-Water Medium.

The experimental results for steel balls in engine oil in three different medium are presented in Figures 4, 5, 6, and 7.

From Figure (4), a decrease in the length (Distance) of the measuring cylinder has a corresponding increase in time (average time) taken by each ball to reach its terminal velocity. A steel ball of size 1.57E-3m was observed as shown in Figure (4), to have gradually moved from its drop point of 0.17 m at a time of 0.00sec to an interfacial time of 3.07 s at a rate of -0.028 m/s and a correlation coefficient (R^2^) of 99.9% before reaching its terminal velocity at the time of 6.18sec. Thus;

(17) d=0.169−0.025t

Where: d = distance moved by ball in meters, t = Average time of ball in Second.

In the same vein, the ball size of 2.53E-3m was equally introduced into the same fluid media to observe its behaviour and it was found as shown in Figure 2, that it took the ball an average time of 1.38sec to reach the interface at a rate of -0.061 m/s with a corresponding correlation (R^2^) of 99.9% and it took the ball an average time of 2.92sec to reach its terminal velocity.

Hence;

(18) d=0.168−0.057t

Also, a ball size of 3.16E-3m diameter recorded an average interfacial time of 0.78 s at a rate of -0.108 m/s and correlation factor (R^2^) of 99.9% with an average terminal velocity time of 1.61sec.

Thus;

(19) d=0.167−0.100t

The ball size of 3.94E-3m attained its interfacial velocity at a time of 0.57 s at a rate of -0.146 m/s and correlation (R^2^) of 99.8% terminating at a velocity-time of 1.23sec.

Hence;

(20) d=0.166−0.1351t

Finally, using a larger steel ball of diameter 8.69E-3m, it was observed as shown in Figure (2), that the ball attained its interfacial velocity at a quicker time of 0.23sec compared to the previously used balls at a rate of -0.372 m/s and coefficient of correlation (R^2^) of 98.2% and attained its terminal velocity at the time of 0.51sec.

Hence;

(21) d=0.169−0.355t

After taking the readings of the last steel ball, the experimental media was then replaced with engine oil-water to study the behaviour of the steel balls. A steel ball of 1.57E-3m was used first as in the previous case and it was observed as shown in Figure (5), that the ball gradually came to an interfacial rest at an average time of 2.92 s at a decreasing rate of -0.029 m/s and a coefficient of correlation (R^2^) of 99.5% before reaching its terminal velocity at an average time of 3.58sec.

Thus;

(22) $$d=0.170+0.033t-0.001t^{2}$$

In the same vein, the steel ball of 2.35E-3m was dropped into the medium and it was observed to reach the fluid interface at an average time of 1.33 s at a rate of -0.064 m/s and R^2^ of 100% before reaching terminal velocity at an average time of 1.75sec.

Thus;

(23) d=0.169−0.063t

Also, the ball size of 3.16E-3m was used and its interfacial impact time was observed to be 0.8 s at a rate of -0.103 m/s and coefficient of correlation (R^2^) of 99.8% with 1.14sec terminal velocity-time.

Hence;

(24) $$d=0.168-0.095t-0.009t^{2}$$

The steel ball size was then increased to 3.94E-3m and dropped into the media. The behaviour of the ball was then recorded as shown in figure (5); it was observed that its interfacial time was reached at an average time of 0.6sec and terminal velocity reached at an average time of 0.89 s at a rate of -0.141 m/s and an R^2^ of 99.9%.

Thus;

(25) $$d=0.169-0.121t-0.032t^{2}$$

And the larger steel ball of diameter 8.69E-3m was introduced into the media and its interfacial impact was quickly reached at an average time of 0.23 s at a rate of -0.212 m/s and coefficient of correlation (R^2^) of 88.5% although at a distance of 0.12–0.08m as seen in figure (5), the steel ball experienced a disturbance before stabilizing towards the interface and finally reaching its terminal velocity at an average time of 0.43sec.

Thus;

(26) $$d=0.177+0.492t-0.747t^{2}$$

Again, the experimental fluid was then replaced this time with the kerosene-glycerol medium to study the behavioural movement of the steel balls in the fluid. As can be seen from figure (6), all the steel balls experienced almost similar movement from drop point of 0.17m–0.14m before diving of time. Ball size of 1.5E-3m attained an interfacial average time of 0.23 s at a rate of -0.394 m/s and a correlation coefficient (R^2^) of 94.6% before reaching its terminal velocity at 2.87sec.

Thus;

(27) $$d=0.172-0.189t-0.923t^{2}$$

Also, ball size 2.35E-3m attained its interfacial average time of 0.2sec and terminal velocity of 1.27 s at a rate of -0.446 m/s and coefficient of correlation (R^2^) of 98.9%.

Hence;

(28) $$d=0.171-0.272t-0.873t^{2}$$

In the same vein, the ball size of 3.16E-3m attained its interfacial time at an average time of 0.17 s at a rate of -0.484 m/s and correlation coefficient (R^2^) of 98.4% with its terminal velocity being reached at the time of 0.87sec.

Thus;

(29) $$d=0.169+0.041t-3.160t^{2}$$

Again the ball size of 3.94E-m was observed to have reached the interface at an average time of 0.14 s at a rate of -0.550 m/s with a terminal velocity time of 0.6sec and correlation (R^2^) of 96.8% (Figure 6).

Thus;

(30) $$d=0.168+0.314t-6.284t^{2}$$

Finally, the ball of diameter 8.69m reached its interfacial time at 0.13 s at a rate of -0.548 m/s and coefficient of correlation (R^2^) of 88.3% and reaching its terminal velocity at 0.3sec.

Hence;

(31) $$d=0.169+0.147t-5.120t^{2}$$

Using the last experimental fluid media of kerosene-water for the steel ball measurement, it was observed as shown in figure (7), except for the ball size 1.5E-3m, all other balls behaved similarly in movement till attaining interfacial rest. The ball of diameter 1.5E-3m attained its interfacial and terminal velocity at an average time of 0.23sec and 0.5sec respectively at a rate of -0.374 m/s with correlation (R^2^) of 99.6%.

Thus;

(32) $$d=0.170-0.079t-1.295t^{2}$$

Both steel ball of diameter 2.35E-3m and 3.16E-3m attained same interfacial and terminal velocity at an average time of 0.17sec and 0.33sec respectively at a rate of -0.470 m/s and correlation (R^2^) of 96.9%.

Thus;

(33) $$d=0.170-0.158t-1.744t^{2}$$

In the same vein, as can be observed in figure (5), ball sizes of 3.94E-3m and 8.69E-3m also attained same interfacial and terminal velocity at an average time of 0.17sec and 0.27sec respectively at a rate of -0.512 m/s and correlation coefficient (R^2^) of 96%.

Hence;

(34) $$d=0.171-0.155t-2.170t^{2}$$

**Figure 8:**
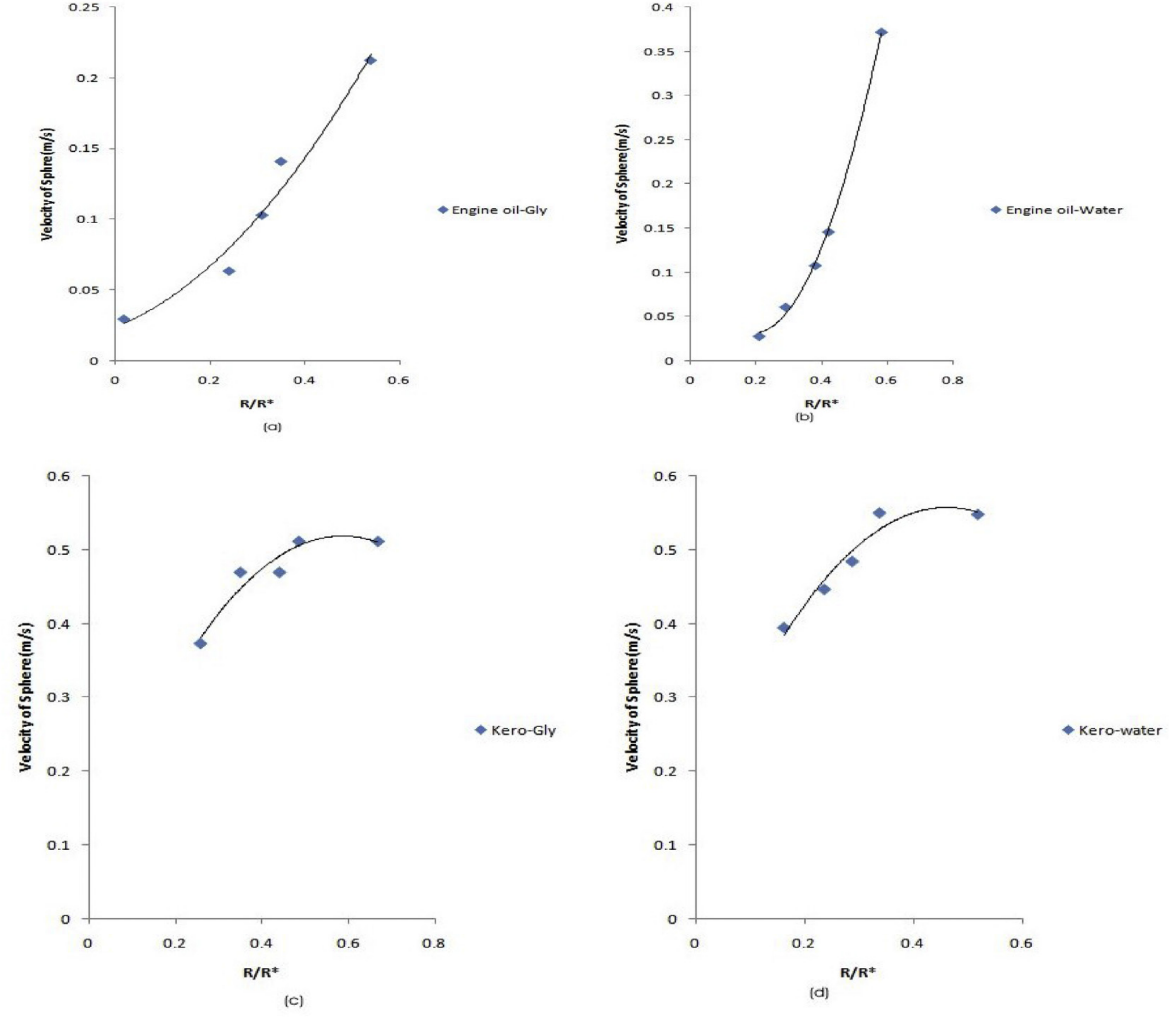
(a–d): Shows the plot of velocity against external ball diameter in the various media.

Similarly, to understand the impact of the various internal ball diameters on the velocity of the balls relative to their external diameters in the fluids, a plot of velocity against ball diameters at the interface of the fluids was studied (see Figure 8).

As observed in figure 8(a-d), an increase in the internal ball diameter results in a corresponding increase in velocity and external diameter. At a correlative coefficient (R^2^) of 99.8%, the velocity of the internal ball increased simultaneously with the external diameter in the engine oil-glycerol mixture (Figure 8a) with;

(35) VEo−Gly=0.113[1+7.611RR∗−19.912RR∗2]

Where; V is the velocity of balls in fluid and D∗(RR∗ ) the external ball diameter.

Also at a correlation of 96.6%, in the engine oil-water mixture figure (8b), the velocity of the ball increased with;

(36) VEo−water=0.024[1+5.542RR∗+17.167RR∗2]

While, in the fluid mixture of kerosene –glycerol figure (8c), at an R^2^ of 94.4%, the velocity of the ball relative to its external diameter was;

(37) Vkero−Gly=0.146[1+12.178RR∗−13.178RR∗2]

And finally, the velocity of the balls in kerosene-water mixture figure (8d), at a correlation of 91.6% is given by;

(38) VKero−water=0.079[1+18.975RR∗−16.165RR∗2]

### 4.2 Theoretical results

**Figure 9:**
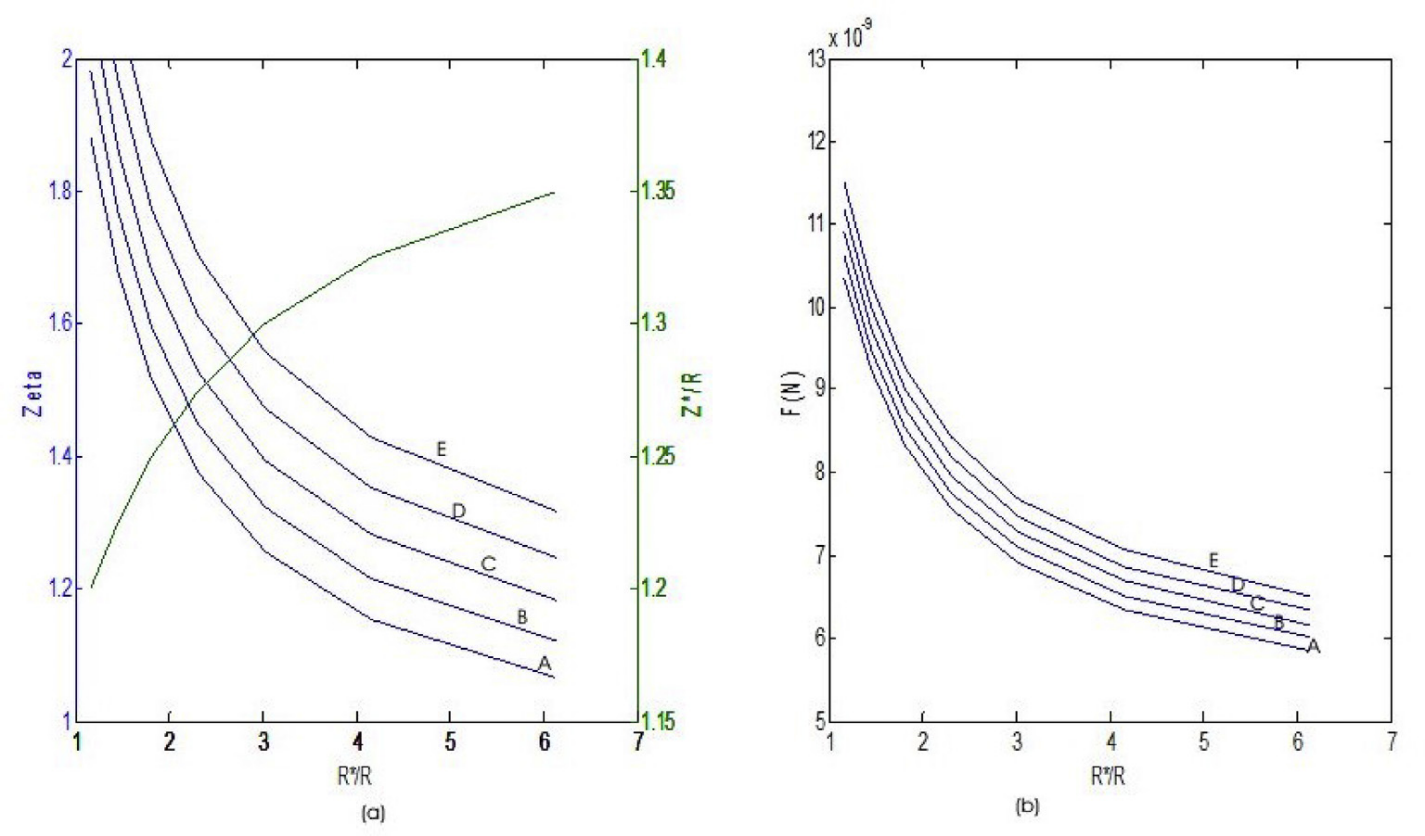
(a–b): shows variations of Zeta ζ & Drag force (F_n_) against external-internal radius ratio of steel balls (a) Zeta & Z/R against R∗/R (b) F_n_ against R∗/R. Where; A, B, C, D and E on the plots, indicates the various ball sizes being analyzed.

In analyzing the forces acting on each ball as it approaches the interface of each fluid, a high-speed computer program (MatLab) tool was employed to evaluate Eqs. (13), (14), and (15) subject to boundary conditions defined in figure (1) to determine zeta ζ and zeta $\zeta^{*}$ which defines the correction factor between the external-internal ball radii relative to the various fluids. The use of MatLab software program seems to give a more precise iteration solution to the model (Eqs. (13), (14), and (15)) and the results presented in figure (9).

Figure (9a), shows that the correction factor ζ decreases with an increase in the external-internal ball radius ratio R∗/R and vice versa while for a series of $\frac{\zeta }{\text{R}}$ which is an external ball β property for the curve-fitting solution (Eq. (12)), for which β < α and β > 0 with α being the internal ball property assumed between 0.45, 0.05 and 0.75. $\frac{\zeta }{\text{R}}$, decreases with a decrease in R∗/R ratio and increases with increase in the ball radius/interface curvature ratio. In the same vein, as can be observed from figure (9b) the drag force (F_n_) on each of the steel balls was also found to decrease with an increase in ball radius ratio R∗/R. Thus, resulting in the following equations for zeta ζ and the drag force *F*_*n*_ for steel ball of diameter 1.5E-3m (line A) as observed in figure (9a), zeta, decreases continuously from point 1.95 till attaining rest and having; computer curve-fit gives:

(39) ζ=2.444[1+0.210R∗R−0.020R∗R2]

Also, ball diameter 2.35E-3m (line B), decreased from point 2.1 to its settling point resulting in

(40) ζ=2.528[1+0.200R∗R−0.019R∗R2]

Ball diameter 3.16E-3m (line C) had

(41) ζ=2.569[1+0.191R∗R−0.018R∗R2]

At point 2.3 as seen in figure (9a), Ball diameter 3.94E-3m (line D) decreased continuously with

(42) ζ=2.622[1+0.185R∗R−0.017R∗R2]

And finally, at point 2.5 the larger steel ball of 8.69E-3m (line E) had a correction factor of

(43) ζ=3.114[1+0.232R∗R−0.023R∗R2]

Similarly, the force (*f*_*n*_) acting on all steel ball was also found to decrease with increasing ball diameter ration figure (9b) and vice versa thus, force response of ball diameter 1.5E-3m was found to be

(44) fn=13.00[1+0.222R∗R−0.023R∗R2]

For ball diameter 2.35E-3m, the force was

(45) fn=12.78[1+0.200R∗R−0.020R∗R2]

Also, a steel ball of diameter 3.16E-3m had a force response of

(46) fn=13.39[1+0.204R∗R−0.021R∗R2]

Whereas, steel ball 3.94E-3m experienced a force effect of

(47) fn=14.19[1+0.216R∗R−0.023R∗R2]

Finally ball diameter of 8.69E-3m had a force response of

(48) fn=14.60[1+0.219R∗R−0.023R∗R2]

### 4.3 Curve-fitted model equation

Correlations obtained from figure (9) as given in Eqs. (39), (40), (41), (42), (43), (44), (45), (46), (47), and (48) are all in terms of $R^{*}/R$. R is ball radius while $R^{*}$is the radius of curvature at the interface. Thus, the ratio $R^{*}/R$ is a large quantity. Its inverse will be more valuable and this informed the choice of Eq. (16) to which all the data of figure (9) were fitted as discussed in Section (2.2).

The constants of Eq. (16) were found to be: A = 0.12246, ∈ = -0.164 and∋ = 8.733.

Eq. (16) now becomes:

(49) $$\zeta =\frac{0.12246}{{\left(1-\frac{R}{z^{*}}\right)}^{-0.164}{\left(1-\frac{R}{R^{*}}\right)}^{8.733}}$$

The form of Eq. (16) was chosen such that if the interface radius denoted by R∗ becomes infinite or z∗ becomes very large, ζ will still be finite. For a deformable interface, just as in this case, a liquid-liquid interface, where the surface of the first liquid z∗ is relatively large and as R is small, R/z∗ ~ 0. Thus, Eq. (49) becomes

(50) $$\zeta =0.12246{(1-\frac{R}{R^{*}})}^{-8.733}$$

By binomial expansion, Eq. (50) becomes

(51) $$\text{ζ}=\;0.12246\left[1+8.733\frac{\text{R}}{\text{R∗}}-4.366{\left(\frac{\text{R}}{\text{R∗}}\right)}^{2}+\cdot \cdot \cdot \right]$$

Substituting Eq. (51) into Eq. (13), one gets

(52) $$\text{Vζ}=0.12246\left[1+8.733\frac{\text{R}}{\text{R∗}}-4.366{\left(\frac{\text{R}}{\text{R∗}}\right)}^{2}+\cdot \cdot \cdot \right]$$

The theoretical expression for velocity (with its correction factor) Eq. (52) is of the same form with the experimental velocity expressions of Eq. (35) for steel ball in engine oil-glycerol media and Eq. (37) for steel balls in kerosene-glycerol media.

### 4.4 Validation of results

In comparison to literature, Stimson and Jeffrey (1926) used continuous differential equations in most of their findings and other researchers used the FORTRAN program in their iteration processes. In this study, a theoretical and experimental approach was used to consider two ball samples, one ball being the main ball inside the arbitrary ball generated by the ball-liquid curvature at the interface as the second ball. This study also used a higher and more accurate program (MATLAB) but has the ability to show the distributions of force and correction factor models on the balls that have not been identified in the literature (see Table 1 and the curve-fitted model equation). The findings of this analysis were consistent with the findings of Stimson and Jeffrey (1926).

**Table 1:** Theoretical and experimental forces for each particle in the various liquids.

Samples	R/R∗	Hydrodynamic Force ($F_{D}=6\pi \mu \mathrm{RV}æ$)Nm	
**Steel Balls**		Theoretical	Experimental
Eng.Oil-Glycerol	0.83	1.95E-5	2.16E-5
Eng.Oil-Water		1.13E-5	1.25E-5
Kero-Glycerol		0.82E-5	0.89E-5
Kero-Water		1.15E-5	1.32E-5

**Figure 10:**
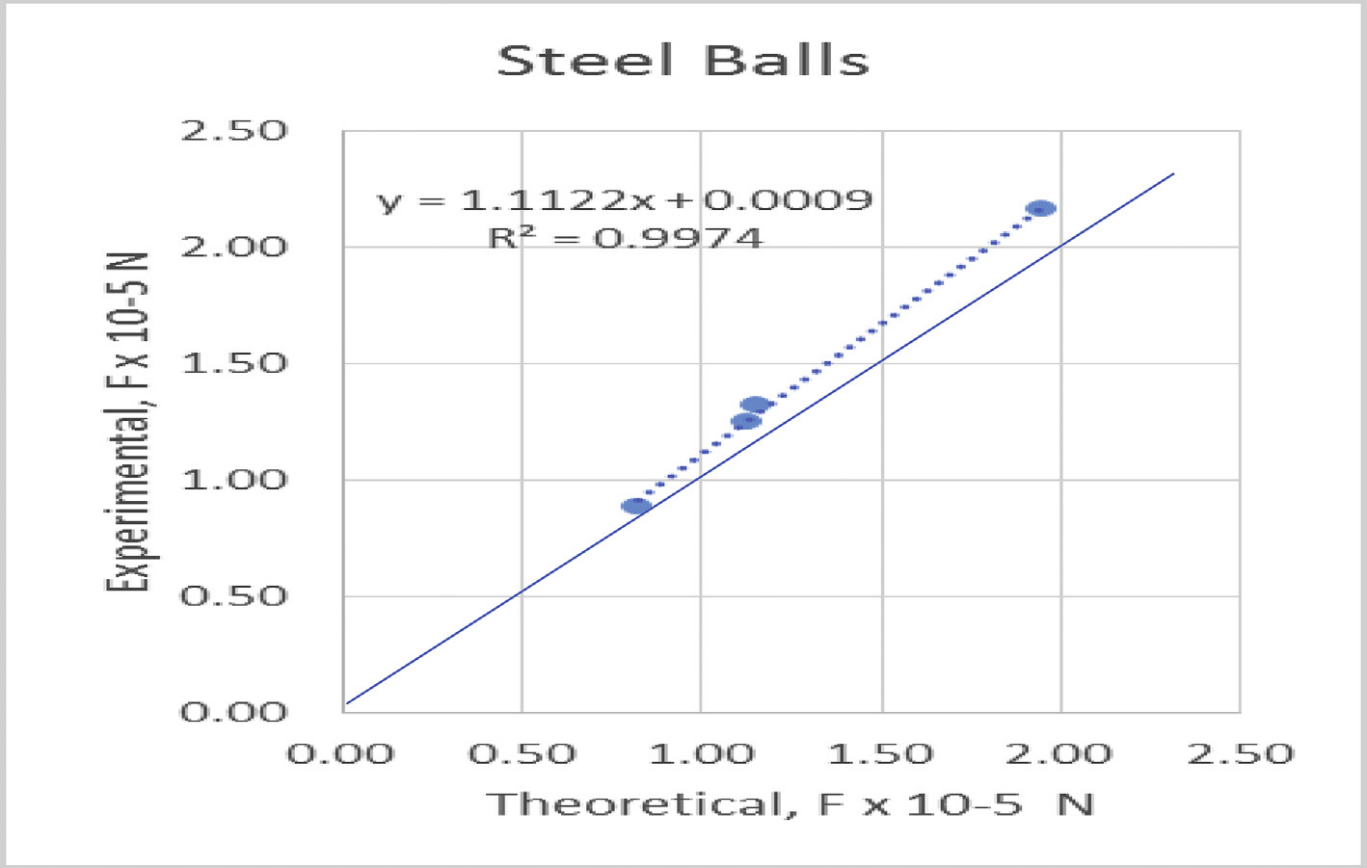
Plot of Experimental Force against Theoretical Forces of the various Balls.

To confirm if the theoretical model assumed was valid, the theoretical and experimentally determined hydrodynamic forces were calculated for the size ratio R/R∗ = 0.83 in all cases. The data are given in table (1) for each of the steel balls in various liquids. The data were equally plotted in figure (10) to view the spread of the data (see Table 2).

**Table 2:** ANOVA summary for steel ball.

Regression Statistics								
Multiple R	0.998681671							
R Square	0.997365081							
Adjusted R Square	0.996047621							
Standard Error	3.37873E-07							
Observations	4							
ANOVA								
	*df*	*SS*	*MS*	*F*	*Significance F*			
Regression	1	8.64217E-11	8.64E-11	757.0365851	0.001318329			
Residual	2	2.28316E-13	1.14E-13					
Total	3	8.665E-11						
	*Coefficients*	*Standard Error*	*t Stat*	*P-value*	*Lower 95%*	*Upper 95%*	*Lower 95.0%*	*Upper 95.0%*
Intercept	8.77017E-09	5.3756E-07	0.016315	0.988464481	-2.30416E-06	2.3217E-06	-2.30416E06	2.3217E-06
X Variable 1	1.11217662	0.04042177	27.5143	0.001318329	0.938255781	1.286097459	0.938255781	1.286097459

The analysis was conducted to strengthen the observed validity of the statistical results using a multi-variant ANOVA and as can be observed from table (2) summary output for steel balls, R^2^ was found to be 0.997 with a significance *F* of 0.001 at 95% confidence level which suggests both theoretical and experimental forces be significantly the same. Thus, agreeing with the statistical results obtained for steel balls. The agreement between the experimental results and those obtained using the theoretical model confirms the validity of the theoretical model.

## 5 Conclusion

Given the different findings studied (Experimental and Theoretical), the theoretical analysis included, first, the modification of Stimson and Jeffrey technique to that of a sphere inside a larger sphere; the larger sphere functions as a curved surface in contact between the two spheres. The theoretical model was solved using a MATLAB program and the correction factor (ζ) to the drag force and hydrodynamic forces obtained. A model equation describing the velocity as a function of the diameter ratio (R/R∗) was obtained from the study. The analyzed results showed that an increase in the reciprocal ratio of the ball diameter (R∗/R) in the different liquids resulted in a corresponding decrease in the correction factor (ζ) which is a significant parameter required to evaluate the hydrodynamic force effect on the balls as they penetrate one liquid medium to the other. This was considered to be comparable to the model equations derived from the experimental tests. The experiment involved the observation and recording of fall of a ball slowly falling on the surface of a liquid pair, the ball slowly falling and encounters the interface separating the two liquids. The ball either remains suspended at the curved interface or falls into the second liquid after some delay. In certain situations, the delay is so small that it cannot be reported. Fall velocities were also calculated from the measurement of the fall distance as a function of time. The mathematical model of the falling velocity as a function of the diameter ratio (R/R∗) was reported equally. The theoretical model findings were compared with the experimental results used to check the validity of the theoretical model. The experimental and theoretical hydrodynamic forces were estimated for a diameter ratio (R/R∗) of 0.83 and the results were compared using the ANOVA model. It was found that the experimental and theoretical findings for steel balls were significantly the same in both analysis techniques. The results of this study were consistent with those of Stimson and Jeffrey (1926). This research is useful in biomechanics in the field of arterial blood flow. The motion of red blood cells through veins or capillaries, as well as the fate of gas bubbles in the bloodstream, which are of great biological and therapeutic importance and potential areas of application of this research. Even, to determine the motion of small particles or macromolecules near permeable surfaces and to determine the concentrations of particles on reverse osmosis, mineral filtration, dialysis or drip irrigation surfaces and other biological applications in which fluid (liquid and gas) moves through membranes or cell walls.

## Declarations

### Author contribution statement

Nduaguba K. C. & Chukwuneke J. L.: Performed the experiments; Analyzed and interpreted the data; Contributed reagents, materials, analysis tools or data; Wrote the paper.

Omenyi S. N.: Conceived and designed the experiments.

### Funding statement

This research did not receive any specific grant from funding agencies in the public, commercial, or not-for-profit sectors.

### Competing interest statement

The authors declare no conflict of interest.

### Additional information

No additional information is available for this paper.
